# Harnessing exercise to combat chronic diseases: the role of Drp1-Mediated mitochondrial fission

**DOI:** 10.3389/fcell.2025.1481756

**Published:** 2025-02-26

**Authors:** Yingxin Sun, Junchen He, Lei Bao, Xiaoming Shi, Jinghong Wang, Qingwen Li

**Affiliations:** ^1^ School of Exercise and Health Sciences, Tianjin University of Sport, Tianjin, China; ^2^ Department of Dermatology, Tianjin Academy of Traditional Chinese Medicine Affiliated Hospital, Tianjin, China; ^3^ Department of Dermatology, Tianjin lnstitute of lntegrative Dermatology, Tianjin, China

**Keywords:** physical exercise, mitochondrial fission, Drp1, chronic diseases, mechanism

## Abstract

Enhanced Drp1 activity mediates excessive mitochondrial fission, contributing to the onset and progression of various chronic diseases, including neurodegenerative, cardiovascular, and metabolic disorders. Studies indicate that exercise mitigates mitochondrial dysfunction by modulating Drp1-related signaling targets, thereby inhibiting Drp1 activity and reducing excessive mitochondrial fission. This, in turn, enhances mitochondrial function and cellular metabolism. This review synthesizes the current understanding of Drp1 structure and activation mechanisms, and analyzes the effects of exercise interventions on Drp1-mediated mitochondrial fission in different disease models to improve common chronic conditions. This research deepens our insight into the specific mechanisms of Drp1-induced excessive mitochondrial fission in chronic disease pathogenesis, offering new theoretical support and practical guidance for exercise as a non-pharmacological intervention strategy.

## 1 Introduction

The rising prevalence of health risk factors, coupled with ageing populations, has contributed to an increased burden of chronic non-communicable diseases (NCDs), including cardiovascular diseases (CVD), neurodegenerative conditions, and metabolic diseases (Disease et al., 2018). With the ongoing advancements in modern medicine, it has become increasingly evident that the onset and progression of chronic diseases are intricately linked to a multitude of factors, among which physical inactivity play a pivotal role in their prevention and management (Lee et al., 2012). In contrast, research indicates that adopting regular moderate physical activity can substantially decrease the risk of developing these chronic diseases (Sagastume et al., 2022; Popa-Wagner et al., 2020; Li Y. et al., 2023).

Mitochondria function as intracellular energy producers, and their dysfunction is intricately associated with the onset and progression of various chronic diseases. In addition to cellular energy supply, mitochondria are signaling organelles with multiple functions such as regulating cellular metabolism (Weinberg et al., 2015), signaling pathways (Shen et al., 2022), stress response (Andrieux et al., 2021) and apoptosis (Harrington et al., 2023). As essential intracellular energy generators and channels that regulate cell death, mitochondrial are meticulously regulated to maintain their quality and quantity (Gan et al., 2018). The dynamic balance of mitochondria, including the fusion and fission processes, in response to metabolic or environmental stresses (Yapa et al., 2021). In this process, mitochondrial fission, mediated by Dynamin-related protein 1 (DRP1), plays a pivotal role. As a GTPase, the function and regulatory role of Drp1 is regulated by post-translational modifications such as phosphorylation, ubiquitination, and deacetylation that affect its activation, localization, and protein interactions. Consequently, these diverse modifications play a crucial role in governing mitochondrial fission and function. Extensive research has indicated a strong correlation between post-translational modifications of Drp1 and prevalent chronic conditions, including neurodegenerative diseases (Bhatti et al., 2023), cardiovascular diseases (Umezu et al., 2020), and metabolic diseases (Kugler et al., 2021). Therefore, an in-depth study of the mechanisms related to Drp1-mediated mitochondrial fission will provide important reference information and research directions for intervening in mitochondrial dysfunction-related diseases.

Exercise serves as an effective intervention by regulating mitochondrial homeostasis through various mechanisms, thereby exerting a positive impact on chronic diseases. Physical exercise has been shown to enhance mitochondrial biogenesis, augment both the quantity and functionality of mitochondria, and preserve the structural integrity of the mitochondrial network. This is achieved through the regulation of the dynamic equilibrium between mitochondrial fusion and fission processes (Hu et al., 2021). Additionally, physical exercise can intervene in chronic diseases triggered by abnormal mitochondrial fission, which is gradually becoming a new strategy to combat these diseases (San-Millan, 2023). Currently, there is inconsistency among study findings, which correlates closely with exercise patterns and disease types. This review delves into the mechanisms of mitochondrial fusion and fission, and the importance of mitochondrial fission in maintaining mitochondrial homeostasis. Additionally, the investigation delves into the impact of Drp1-mediated mitochondrial fission abnormalities on the development of chronic diseases, while also examining the influence of exercise on modulating mitochondrial fission to slow these conditions.

## 2 Mechanisms of mitochondrial fusion and fission

**FIGURE 1:**
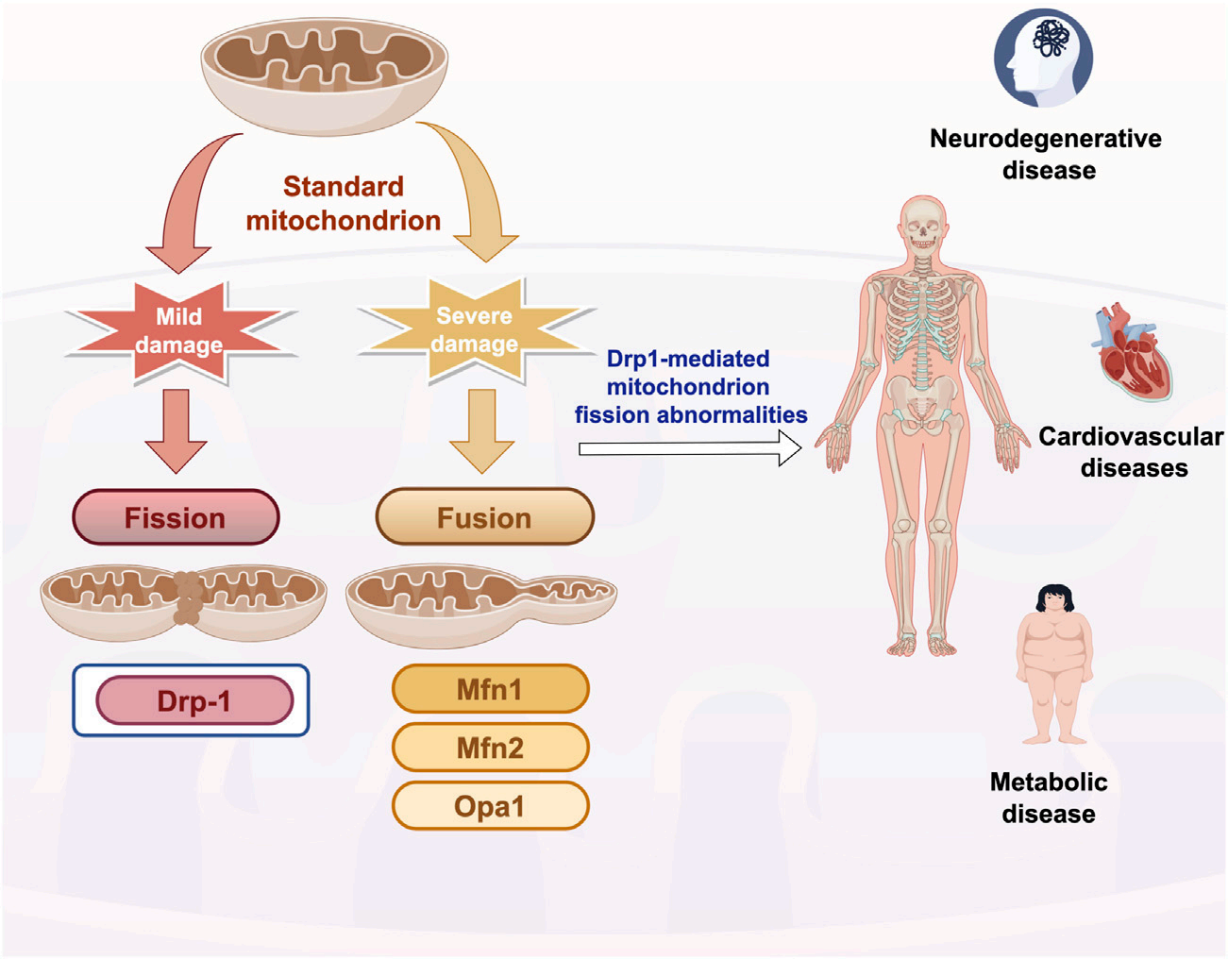
Overview of mitochondrial fusion and fission mechanisms Drp-1: dynamin-related protein 1; Mfn1/2: Mitofusin1/2. Opa1: Option Atrophy 1; Quotes from the Researcher’s House. Export ID:RITUT27024.

Mitochondrial homeostasis, encompassing the processes of fusion and fission, constitutes a crucial mechanism by which cells preserve functionality and adapt to stress. Research has demonstrated that, in instances of mild damage, mitochondria undergo fission to isolate affected regions, thereby limiting the propagation of cellular damage. When mitochondria experience significant damage, the fusion machinery is activated to preserve the integrity of the mitochondrial network (Eisner et al., 2018). Mitochondrial fusion and fission are facilitated by a series of large GTPase family members, which are proteins highly conserved across yeast, *Drosophila*, and mammals (Youle and van der Bliek, 2012). Mitochondrial fusion is facilitated by the outer membrane fusion proteins Mitofusin 1 and 2 (Mfn1/2), along with the inner membrane fusion protein Optic Atrophy 1 (Opa1). In contrast, mitochondrial division is predominantly mediated by the cytoplasmic protein Drp1 (Dynamin-related protein 1) (Youle and van der Bliek, 2012). During mitochondrial division, Drp1 is recruited to the mitochondrial surface, where it assembles into a helical structure and undergoes contraction, ultimately leading to the severance of both the inner and outer membranes. Abnormalities in Drp1-mediated mitochondrial fission are critically implicated in the pathogenesis of numerous chronic diseases. Aberrant activation of Drp1 results in mitochondrial dysfunction, apoptosis, and metabolic disorders, thereby exacerbating disease progression. Consequently, targeting Drp1 and its regulatory pathways may represent a novel strategy for the intervention and management of chronic diseases (Figure 1).

## 3 Molecular mechanisms of Drp1 activation and mitochondrial fission

Mitochondrial division is a critical process for preserving mitochondrial function and ensuring quality control within cells, primarily operating through two distinct mechanisms: medial division (intermediate of mitochondria) and peripheral division (within 25% of the mitochondrial edge) (Kleele et al., 2021). In the mouse cardiomyocytes, the presence of two distinct modes of division has been observed, alongside the identification of distinct protein mechanisms that autonomously regulate these modes (Kleele et al., 2021). Medial divisions predominantly occur within the central region of mitochondria and are primarily linked to regular cellular growth and metabolic demands. Peripheral divisions mainly happen at the ends of mitochondria and are linked to damage or stress. The resulting small mitochondria typically lack mtDNA and mtRNA, making them more prone to lysosomal degradation, which helps eliminate damaged mitochondria. The two modes of mitochondrial division serve distinct functions in cellular physiological and pathological processes, with post-translational modifications (PTMs) of Drp1 being pivotal to these mechanism (Purkanti and Thattai, 2015).

### 3.1 Phosphorylation of Drp1

Research has demonstrated that the regulation of mitochondrial division by Drp1 is modulated by PTM, notably the phosphorylation status of Drp1. Phosphorylation at two key serine sites, S616 (Gao et al., 2022) and S637 (Hu et al., 2023), is pivotal in modulating Drp1’s activity. The phosphorylation of Drp1 at serine 616 augments its activity, facilitating its translocation from the cytoplasm to the outer mitochondrial membrane and its interaction with receptor proteins such as Mff and Fis1 (Parida et al., 2023). This process induces mitochondrial division in the medial region, a phenomenon typically associated with normal cellular growth and metabolic requirements, thereby contributing to the equitable distribution of mitochondria within the cell (Chang et al., 2022). Phosphorylation at serine 637 resulted in the inhibition of Drp1 activity and decreased its localization to the outer mitochondrial membrane, thereby impeding mitochondrial fission (Sun et al., 2024). In the context of peripheral division, the phosphorylation of Ser637 may serve a protective role for mitochondria against excessive division by inhibiting the overactivation of Drp1. This mechanism thereby contributes to the preservation of mitochondrial network integrity (Sun et al., 2024). However, recent research indicates that the phosphorylation of Ser637 may indirectly facilitate the phosphorylation of Ser616 via a complex mechanism, thereby modulating mitochondrial division (Valera-Alberni et al., 2021). For instance, under ischemia-hypoxia conditions, there is a notable increase in the phosphorylation of Ser637, which is subsequently followed by a significant elevation in the phosphorylation of Ser616. This observation implies that Ser637 may indirectly facilitate the phosphorylation of Ser616 through an underlying mechanism (Duan et al., 2023).

### 3.2 Regulatory mechanisms of phosphorylation

The phosphorylation of Drp1 is finely regulated by multiple protein kinases. The activity of Drp1 is mainly regulated by phosphorylation at Ser616 and Ser637. The Cyclin B1/Cyclin-dependent kinase 1 (Cyclin B1/Cdk1) complex phosphorylates the Ser616 site of Drp1 and promotes its translocation in mitochondria (Cai P. et al., 2024). Similarly, Protein kinase B (Akt) promotes mitochondrial fission by directly phosphorylating the S616 site of Drp1 (Liao et al., 2024). Furthermore, PINK1 (PTEN-induced kinase 1) has been demonstrated to phosphorylate Drp1 at the Ser616 residue, thereby modulating mitochondrial fission. This process subsequently influences neuronal synaptic development and plasticity (Gao et al., 2022). Conversely, phosphorylation at Ser637 inhibited the activity of Drp1 and reduced its accumulation in the outer membrane of mitochondria, thus inhibiting mitochondrial fission. For instance, AMP-activated protein kinase (AMPK) increases the phosphorylation of Ser637 on Drp1, which inhibits mitochondrial fission, and it also prevents alterations in the morphology of the ER and mitochondria (Hu et al., 2024). In a similar way, protein kinase A (PKA) inhibits the GTPase activity of Drp1 by directly phosphorylating the S637 site at Drp1, thereby preventing mitochondrial fission and preserving the integrity of the mitochondrial structure (Cai P. et al., 2024). Additionally, Calcineurin dephosphorylates Drp1 at Ser637 and inhibits its activity, thereby reducing mitochondrial fission, a process that plays an important role in cellular stress and injury. This conclusion was confirmed in a mouse model of hippocampal injury caused by hepatic ischemia-reperfusion. Calcineurin activation led to Drp1 dephosphorylation and its movement to mitochondria, worsening mitochondrial fission and neuronal apoptosis. Treatment with the Calcineurin inhibitor FK506 increased Drp1 phosphorylation, prevented its mitochondrial translocation, and reduced mitochondrial fission (Yu et al., 2019).

### 3.3 Ubiquitination, SUMOylation, and OGlcN acylatio of Drp1

In addition to phosphorylation, Drp1 is regulated by other post-translational modifications such as ubiquitination, SUMOylation, and O-GlcNAcylation, which collectively fine-tune Drp1 activity and mitochondrial fission.

Ubiquitination modification of Drp1 Ubiquitination of Drp1 mainly occurs on its lysine residues, with key sites identified as Lys38 and Lys616, with Lys38 being more commonly ubiquitinated (Li D. et al., 2024). Membrane-Associated RING-CH 5 (MARCH-V), an E3 ubiquitin ligase situated in the outer mitochondrial membrane, selectively targets Drp1 by interacting with Lys38 on the protein (Verhoeven et al., 2023). This interaction enables MARCH-V to transfer ubiquitin to Drp1, marking it for degradation and thereby negatively regulating Drp1’s activity (Karbowski et al., 2007).

SUMOylation modification of Drp1 Unlike ubiquitination, SUMOylation does not mark the protein for degradation, but can modify its function or position at variable regions of Drp1. Among them, SUMO1 coupling promotes the binding of Drp1 to the mitochondrial membrane, thereby enhancing mitochondrial fission (Wasiak et al., 2007). In contrast, SUMO2 and SUMO3 decrease this binding, attenuating mitochondrial fission (Guo et al., 2013). The intricate regulation of SUMOylation on Drp1 depends on the specific cytoplasmic conditions. This modification is crucial during cellular stress, like oxidative stress or hypoxia, as SUMOylation regulates Drp1 activity to maintain mitochondrial balance (Das and Chakrabarti, 2024).

O-GlcNAc glycosylation modification of Drp1 O-GlcNAcylation represents a dynamic post-translational modification that modulates protein function, stability, and subcellular localization. OGlcN acylation of Drp1 is particularly pronounced in both cultured neonatal cardiomyocytes and type 2 diabetic mouse hearts (Gawlowski et al., 2012). Research indicates that O-GlcNAc modification potentially regulates Drp1 activity and mitochondrial fission by influencing its phosphorylation state or interacting synergistically with other post-translational modifications (Li D. et al., 2024). Notably, OGlcN acylation of Drp1 appears to have a contributory effect on the increase in Ser616 phosphorylation and subsequent translocation of Drp1 to mitochondria (Hu et al., 2020).

**FIGURE 2:**
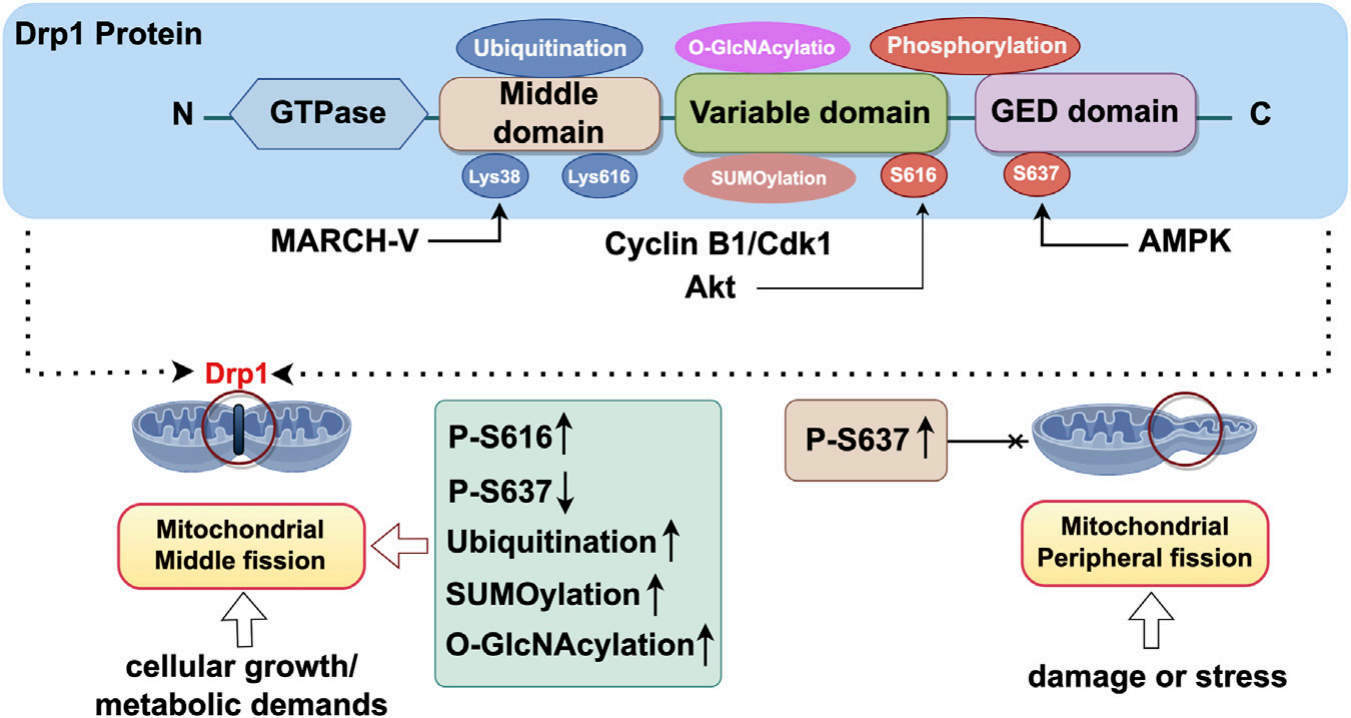
Structure of Drp1 and its activation site. Export Drp1 is a large GTPase belonging to the dynamin superfamily, primarily involved in the regulation of mitochondrial fission. The structural composition of Drp1 includes the GTPase domain, middle domain, GTPase effector domain (GED), and variable domain. The GTPase domain of Drp1 is responsible for the catalytic binding and hydrolysis of GTP. The binding and hydrolysis of GTP are crucial steps in the function of Drp1, driving its translocation from the cytoplasm to the outer mitochondrial membrane and facilitating mitochondrial fission (Rochon et al., 2024). The middle domain serves as a linker between the GTPase domain and the GTPase effector domain (GED) and plays a significant role in the self-assembly process of Drp1. The GTPase effector domain (GED) acts in concert with the GTPase domain to drive the self-assembly and helical polymerisation of Drp1. The variable domain (VD), composed of 136 intrinsically disordered amino acid residues, confers lipid-sensing capabilities to Drp1, regulating its assembly and activity. CyclinB1/Cdk1:Cyclin B1/Cyclin-dependent kinase 1; MARCH-V:Membrane-Associated RING-CH 5; AMPK: AMP-activated protein kinase. Export ID: IAIPTf899f.

In summary, Drp1 activity is regulated by a multitude of intricate factors. Given the diversity of mitochondrial fission, it is challenging for a single post-translational modification to fully account for its effects on both physiological and pathological conditions. Conversely, multiple post-translational modifications of Drp1 may have synergistic or cumulative effects. Consequently, simultaneous assessment of multiple post-translational modifications in a specific cellular environment can provide insight into the relative objectivity of mitochondrial division activation, which is thought to be a major determinant of functional outcome (Figure 2).

## 4 Aberrant modification of Drp1 and chronic disease

Under conditions of cellular stress, the activity of Drp1 is markedly upregulated by PTM, resulting in excessive mitochondrial fission. This phenomenon is intricately associated with various mechanisms of cellular damage and apoptosis (Forrester et al., 2020). For instance, oxidative stress enhances the phosphorylation of Drp1 at the Ser616 residue, resulting in excessive mitochondrial fission, which subsequently intensifies apoptosis (Chang et al., 2023). Furthermore, mitochondrial fission induced by Drp1 constitutes a crucial early event in the cascade leading to neuronal apoptosis in the context of ischemic/hypoxic injury (She et al., 2025). Significantly, dysregulation of Drp1-mediated mitochondrial fission is closely associated with a spectrum of chronic non-communicable diseases (Kleele et al., 2021), including neurodegenerative diseases (Wu et al., 2017; Medala et al., 2021; Oliver and Reddy, 2019), cardiovascular diseases (Umezu et al., 2020; Huan et al., 2023), and metabolic disorders (Wada and Nakatsuka, 2016).

### 4.1 Neurodegenerative diseases

Mitochondria play a pivotal role in orchestrating cellular energy metabolism and maintaining calcium homeostasis. Impairment of mitochondrial function is intrinsically linked to the pathogenesis of numerous neurodegenerative disorders (Bertholet et al., 2016), including Alzheimer’s disease (AD), Parkinson’s disease (PD), and Huntington’s Disease (HD). Notably, Drp1 may be involved in the processes that cause mitochondrial fragmentation and abnormal mitochondrial dynamics in neurodegenerative diseases (Reddy et al., 2011).

AD is a neurodegenerative disorder marked by memory loss and cognitive decline, with key features being extracellular β-amyloid (Aβ) deposits and intracellular neurofibrillary tangles from Tau protein hyperphosphorylation (Liang et al., 2025). Early AD is distinguished by oxidative stress and ER stress as its distinguishing metabolic features (Placido et al., 2015; Aliev et al., 2014). Notably, mitochondrial dysfunction is well-established as a potential driver of the above events and is a prime therapeutic target for early AD lesions (Morton et al., 2021). Basic experiments have also confirmed that mitochondrial dysfunction is an early event in AD (Hauptmann et al., 2009). In the hippocampi of AD mouse models, increased levels of fission proteins like Drp1 and Fis1 and decreased levels of fusion proteins like Mfn1, Mfn2, and Opa1 were observed, suggesting disrupted mitochondrial dynamics in these models (Kandimalla et al., 2018; Manczak et al., 2018). Recent evidence underscores the crucial role of mitochondrial dysfunction, particularly abnormal fission by Drp1, in AD development and progression (Guo et al., 2018). Excessive mitochondrial fission, driven by Drp1, is a hallmark of AD, leading to β-amyloid precursor protein cleaving enzyme 1 (BACE1) activation and Aβ accumulation in the N2a cell AD model transfected with the mutant β-amyloid precursor protein (AβPP) gene (Reddy et al., 2018). Mdivi-1, a Drp1 inhibitor, has been shown to significantly improve learning and memory in APP/PS1 mice, by preventing mitochondrial fragmentation, reducing BACE1 expression and Aβ deposition in the brain (Baek et al., 2017), highlighting Drp1’s key role in Aβ production.

PD is the second most common fast-progressing neurodegenerative disorder, affecting over 6 million people worldwide (Dorsey et al., 2018). It is mainly marked by impaired automatic movement control, like balance and gait, due to degeneration of dopaminergic neurons in the substantia nigra pars compacta of the midbrain (Ponsoni et al., 2023). The pathological mechanism of this condition encompasses the abnormal aggregation of α-synuclein (α-syn), mitochondrial dysfunction, and the generation of oxidative stress (Ye et al., 2023). In transgenic murine models, α-syn pathology is correlated with alterations in Drp1 functionality and aberrant mitochondrial morphology (Banerjee et al., 2022). Research indicates that abnormal α-synuclein aggregation activates Drp1, promoting its phosphorylation at Ser616 and increasing mitochondrial fission. This excessive division damages mitochondrial integrity, leading to dysfunction that impacts neuronal energy metabolism and synaptic function (Portz and Lee, 2021). Furthermore, α-synuclein induces mitochondrial fragmentation through a Drp-1-dependent mechanism, while overexpression of the fusion protein Opa-1 counteracts this fragmentation and cytotoxicity. Drp1-mediated abnormal mitochondrial division is a critical factor in dopaminergic neuronal apoptosis. Knocking down Drp1 inhibits this aberrant division and apoptosis, leading to significant symptomatic improvements in PD mouse models (Zhang et al., 2019).

In HD, the mutant Huntington protein (mHtt) interacts with Drp1, stimulating its activity and causing excessive mitochondrial fission and distribution abnormalities, which in turn disrupt axonal transport and contribute to synaptic degeneration (Sawant et al., 2021). Mitochondrial dysfunction, resulting from the aberrant activation of Drp1 by mutant huntingtin (mHtt), not only impairs neuronal energy metabolism but also exacerbates neuronal damage through the induction of oxidative stress and disruption of calcium homeostasis.

### 4.2 Cardiovascular diseases (CVDs)

As a major energy-demanding organ, the heart is extremely dependent on mitochondrial energy supply to maintain cardiac contraction and cardiomyocyte metabolism (Serasinghe and Chipuk, 2017). Accumulating evidence indicates that disruptions in mitochondrial dynamics may result in inadequate myocardial energy supply, serving as a significant mechanism contributing to the increased incidence of CVDs (Cho et al., 2013). Mitochondrial fission is crucial for cellular homeostasis, but in cardiovascular disease, there is often increased fission and reduced fusion (Forte et al., 2021). This indicates a strong link between Drp1-driven mitochondrial fission and impaired mitochondrial dynamics. Consequently, regulating abnormal mitochondrial fission could be an effective strategy for reducing the risk of coronary heart disease (CHD), hypertension and heart failure.

CHD is heart disease caused by ischemia, hypoxia, or necrosis of the myocardium due to coronary atherosclerosis. Myocardial ischemia and myocardial infarction, resulting from coronary atherosclerosis, can induce myocardial cell damage and necrosis, ultimately leading to diminished cardiac function and heart failure (Ajoolabady et al., 2024). CHD is the result of atherosclerosis involving the coronary arteries, with studies indicating that excessive Drp1 activation in atherosclerosis causes mitochondrial fission, increased reactive oxygen species, and impaired mitochondrial function, ultimately resulting in VSMCs necroptosis and vulnerable plaque formation (Li X. et al., 2024). Research suggests that mitochondrial fission proteins like Drp1 and Mff become more active in cardiac hypertrophy and heart failure, while the fusion protein Opa1 activity diminishes, indicating an imbalance towards excessive fission within cardiomyocytes (Adaniya et al., 2019). Furthermore, Mfn2 expression is downregulated in mouse hearts 1 and 3 weeks after aortic constriction,as well as in 10-month-old spontaneously hypertensive rats with cardiac hypertrophy (Fang et al., 2007). A separate investigation revealed that in both humans and dogs with heart failure, cardiomyocytes display reduced levels of Mfn2 and Opa1, alongside an increase levels of Drp1 and Fis1 (Sabbah et al., 2018). This evidence collectively points to a dysregulation of mitochondrial dynamics characterized by an imbalance towards fission in the pathophysiology of heart disease.

### 4.3 Metabolic diseases

Metabolic diseases are a group of disorders characterized by hyperglycemia, obesity, insulin resistance (IR), and dyslipidemia (Hu and Jia, 2021). Mitochondria, as highly sensitive organelles, undergo fusion, fission and metabolic adaptations in response to changes in the internal and external environment. Studies indicate that mitochondrial dysfunction induces metabolic disorders, leading to the development of metabolic diseases including type 2 diabetes mellitus (T2DM) and obesity (Wada and Nakatsuka, 2016; Pinti et al., 2019).

T2DM is a progressive metabolic disorder that arises from compromised insulin responsiveness in specific target tissues, including the liver (Ling et al., 2020), skeletal muscle (Liu et al., 2014), and adipose tissue (Boengler et al., 2017), alongside inadequate insulin synthesis by pancreatic β-cells (L'Heveder and Nolan, 2013). Accumulating evidence indicates abnormal mitochondrial function is a key mechanism for pancreatic β-cell dysfunction. This dysfunction is strongly associated with reduced mitochondrial membrane potential and expression levels of key regulators of mitochondrial dynamics such as Opa1 or Mfn (Hong et al., 2018). Endothelial dysfunction in diabetes has been linked to altered mitochondrial dynamics, particularly increased fission, which is a significant contributor to this dysfunction (Shenouda et al., 2011). Studies have demonstrated that silencing the Fis1 or Drp1 genes can protect against high glucose-induced inhibition of endothelial-type nitric oxide synthase (eNOS) activity, which is hypothesized to be achieved by reducing mitochondrial reactive oxygen species (ROS) production (Kluge et al., 2013). Clinical studies have shown that the mitochondrial dynamics of diabetic patients are significantly impaired, in which Mfn2 is involved in mitochondrial fusion and is inhibited in the muscles of obese or T2DM patients (Sebastian et al., 2012). In the ApoE-knockout diabetic mouse model, Drp1-mediated mitochondrial fission inhibition through AMPK activation improves endothelial dysfunction and delays atherosclerosis (Wang et al., 2017; Liu et al., 2019). Further studies confirmed the reduction of mitochondrial fission in diabetic mice using a potent and selective Drp1 inhibitor (Wang et al., 2017), indicating that Drp1-targeted inhibition of mitochondrial fission could be a promising therapeutic strategy for managing vascular complications in diabetic patients.

The study revealed that Resistin gene humanized mouse, after 3 months of high-fat diet, there was an increase in the number of fragmented and shortened mitochondria in skeletal muscle, resulting in reduced ATP production and mitochondrial dysfunction. Moreover, the inhibition of mitochondrial division has been shown to enhance muscle insulin signaling and overall insulin sensitivity in obese mice (Jheng et al., 2012). The presented data provide compelling evidence suggesting that Drp1-mediated mitochondrial fission may contribute to the development of insulin resistance. Consequently, targeting the upstream mechanisms of Drp1 also emerges as a potential therapeutic target for obesity-related metabolic diseases.

## 5 Exercise strategies improve Drp1-mediated chronic diseases

Systematical exposure to recurring exercise stimuli results in long-term adaptations of various tissues and induces a myriad of well-known exercise effects, such as increased vascularization and mitochondrial biogenesis, improved cardiac and immune cell function, and enhanced substrate handling by adipose and liver tissue (Walzik et al., 2024). Exercise training has been shown to improve Drp1-mediated chronic diseases by modulating various signaling pathways and molecular targets. The World Health Organization has recently reported that 27.5% of adults worldwide fail to meet the recommended physical activity guidelines (World Health, 2020). Prolonged periods of physical inactivity have been linked to an increased risk of developing type 2 diabetes (Kivimaki et al., 2019), cardiovascular events (Pandey et al., 2015), and all-cause mortality (Kodama et al., 2009). A sedentary lifestyle results in diminished mitochondrial function, while engaging in moderate exercise enhances mitochondrial function and mitigates Drp1-mediated mitochondrial fission (Chang et al., 2022). Research indicates that moderate aerobic exercise can lower Drp1 and Fis1 levels and boost Mfn1/2 and OPA1 levels, helping to balance mitochondrial dynamics (Strain et al., 2024). We conducted a thorough analysis of relevant studies to elucidate how exercise regulation affects mitochondrial fission abnormalities in common chronic diseases, focusing on Drp1 activation.

### 5.1 Regulation of Drp1 activity by exercise

Exercise interventions modulate Drp1 activity and curtail excessive mitochondrial fission via multiple mechanisms. Specifically, aerobic exercise diminishes mitochondrial fission by engaging AMPK and SIRT1 signaling, which inhibits Drp1 phosphorylation at Ser616, thus reducing its activity (Pedrera et al., 2025). Moreover, In an Alzheimer’s disease model, aerobic exercise suppresses Drp1 activity via miR-34a upregulation, decreasing excessive mitochondrial fission, enhancing mitochondrial function, and mitigating neuronal injury (Liu et al., 2024).

### 5.2 Exercise against neurodegenerative disease

Exercise, including aerobic and resistance training, is a potent non-pharmacological strategy against neurodegenerative diseases, reducing disease markers, fostering neurogenesis, and boosting cognitive abilities. Public health efforts should focus on exercise programs for the elderly to prevent AD, PD, and HD (Gutierre et al., 2024). Moderate exercise is beneficial for cognition and AD risk reduction (Valenzuela et al., 2020). Both aerobic and resistance training are effective for cognitive enhancement in older adults, with additional benefits from cognitive-motor dual-task training (Akalp et al., 2024; Montero-Odasso et al., 2023). Aerobic exercise stabilizes sensorimotor network progression in PD patients, improving cognitive functions, and high-intensity treadmill training enhances gait in mild to moderate PD (Johansson et al., 2022; Duchesne et al., 2015). Additionally, home-based exercise significantly improves motor function in HD patients (Al-Wardat et al., 2022).

PGC-1α/Drp1 pathway Research suggests that decreased PGC-1α is associated with the onset of various neurodegenerative diseases, and its upregulation in the hippocampus is crucial for preserving neuronal mitochondrial homeostasis by reducing excessive fission and promoting fusion (Panes et al., 2022). Therefore, upregulation of PGC-1α expression may be an effective therapeutic strategy. Moderate-intensity treadmill exercise in rats with Aβ1-42-induced impairment has been demonstrated to improve spatial learning and memory, likely due to the activation of AMPK and elevated PGC-1α levels in skeletal muscle. The role PGC-1α is pivotal as it directly suppresses Drp1, decreasing mitochondrial fission and fostering mitochondrial biogenesis and functionality (Buler et al., 2014). In essence, exercise positively impacts neurodegenerative diseases by regulating the PGC-1α/Drp1 pathway, enhancing mitochondrial function, and reducing excessive mitochondrial division, supporting its use as a treatment.

### 5.3 Exercise against cardiovascular diseases

Exercise modulates mitochondrial functionality via multifaceted mechanisms, mitigates oxidative stress and apoptosis, thereby exerting a protective influence against cardiovascular pathogenesis. For instance, High-intensity interval training (HIIT) significantly increased mitochondrial respiratory capacity and muscle strength in both young and elderly subjects, ameliorating the age-related decline in cardiomyocytes mitochondrial function (He et al., 2024). Endurance exercise, such as swimming, has been shown to elicit alterations in the mitochondrial life cycle, including modifications in Drp1-mediated mitochondrial fission signaling, thereby enhancing mitochondrial function in cardiomyocytes (Moore et al., 2019).

MiR-30B/p53/Drp1 pathway MiR-30B, a non-coding RNA, inhibits p53 expression, which in turn reduces Bax levels and alleviates cardiomyocyte apoptosis (Hong et al., 2024). In addition, MiR-30B can also reduce the Bax mediated apoptosis by inhibiting the activity of Drp1, reducing mitochondrial fission and maintaining the health of mitochondria (Li SN. et al., 2023). Elevated cardiomyocyte apoptosis serves as a significant indicator of the progression of chronic cardiovascular diseases and is intricately associated with various pathological processes, such as myocardial ischemia, myocardial infarction, heart failure, and atherosclerosis (Cai K. et al., 2024). Exercise potently elevates the expression of miR-30B, which in turn represses the levels of p53 and Drp1 proteins. This regulatory mechanism contributes to the preservation of mitochondrial homeostasis within cardiomyocytes, curbing excessive mitochondrial fission and thus mitigating cardiomyocyte apoptosis (Hong et al., 2024). In addition, aerobic exercise confers cardioprotection against myocardial ischemia-reperfusion injury by modulating mitochondrial dynamics—upregulating Mfn1 and Mfn2 expression and downregulating Drp1 to adjust the fission-fusion balance, thereby reducing reducing myocardial cell apoptosis (Ghahremani et al., 2018).

mTORC1/Drp1 pathway Mammalian target of rapamycin complex 1 (mTORC1) is a major regulatory molecule of cell growth and metabolism, which is involved in the regulation of protein synthesis, lipid synthesis, and autophagy, among which the activation of mTORC1 can inhibit mitophagy (Shimobayashi and Hall, 2014). In addition, Drp1 activation inhibits Parkin expression via Clec16a, reducing mitochondrial autophagy in ischemic vessels (Pedrera et al., 2025). It has been proven that aerobic exercise can inhibit the activity of mTORC1 by activating AMPK (AMP-activated protein kinase), thereby promoting mitophagy to remove damaged mitochondria. In models of heart failure, aerobic exercise reduces mitochondrial fission, improves mitochondrial function, and reduces cardiomyocyte injury by regulating mTORC1/Drp signaling (Chung et al., 2024).

### 5.4 Exercise against metabolic diseases

Currently, both aerobic exercise and resistance training are recommended as first-line exercise therapies for T2DM. Aerobic exercise, which increases maximal oxygen uptake (Boule et al., 2003), reduces obesity (Khalafi et al., 2023)and improves blood pressure (Xi et al., 2021), is an effective strategy to increase the body’s energy requirements and simultaneously enhance insulin sensitivity and fat oxidation. Muscle is one of the most important target tissues of insulin, and muscle weakness and low muscle mass are common in patients with T2DM. A recent study has discovered that after 12 weeks of isolated centrifugal and concentric knee extensor resistance training among healthy elderly males aged 60–76, and centrifugal exercise training was found to exert a significant effect in improving insulin sensitivity, making it an effective approach for the prevention and treatment of T2DM (Chen et al., 2017). Clinical trials have shown that aerobic interval training improves glycemic control to a greater extent in patients with T2DM (Mitranun et al., 2014). It was confirmed that aerobic exercise reduced the levels of Fis1 and Parkin in skeletal muscle of sedentary adults, while increasing the ratio of fusion to fission proteins and promoting a more integrated tubulin network (Axelrod et al., 2019). These alterations were positively associated with the enhanced insulin sensitivity and substrate utilization observed after exercise training. Another study demonstrated that a 12-week aerobic exercise significantly reduced body weight, improved peripheral insulin sensitivity and fat oxidation in obese adults and was positively correlated with reduced levels of phosphorylation at the Ser616 site of Drp1 (Fealy et al., 2014). This confirms that Drp1-mediated mitochondrial fission is reduced after exercise training, suggesting that the downregulation of Drp1 activity may play a crucial role in enhancing insulin resistance in skeletal muscle.

## 6 Perspectives for future studies

**FIGURE 3:**
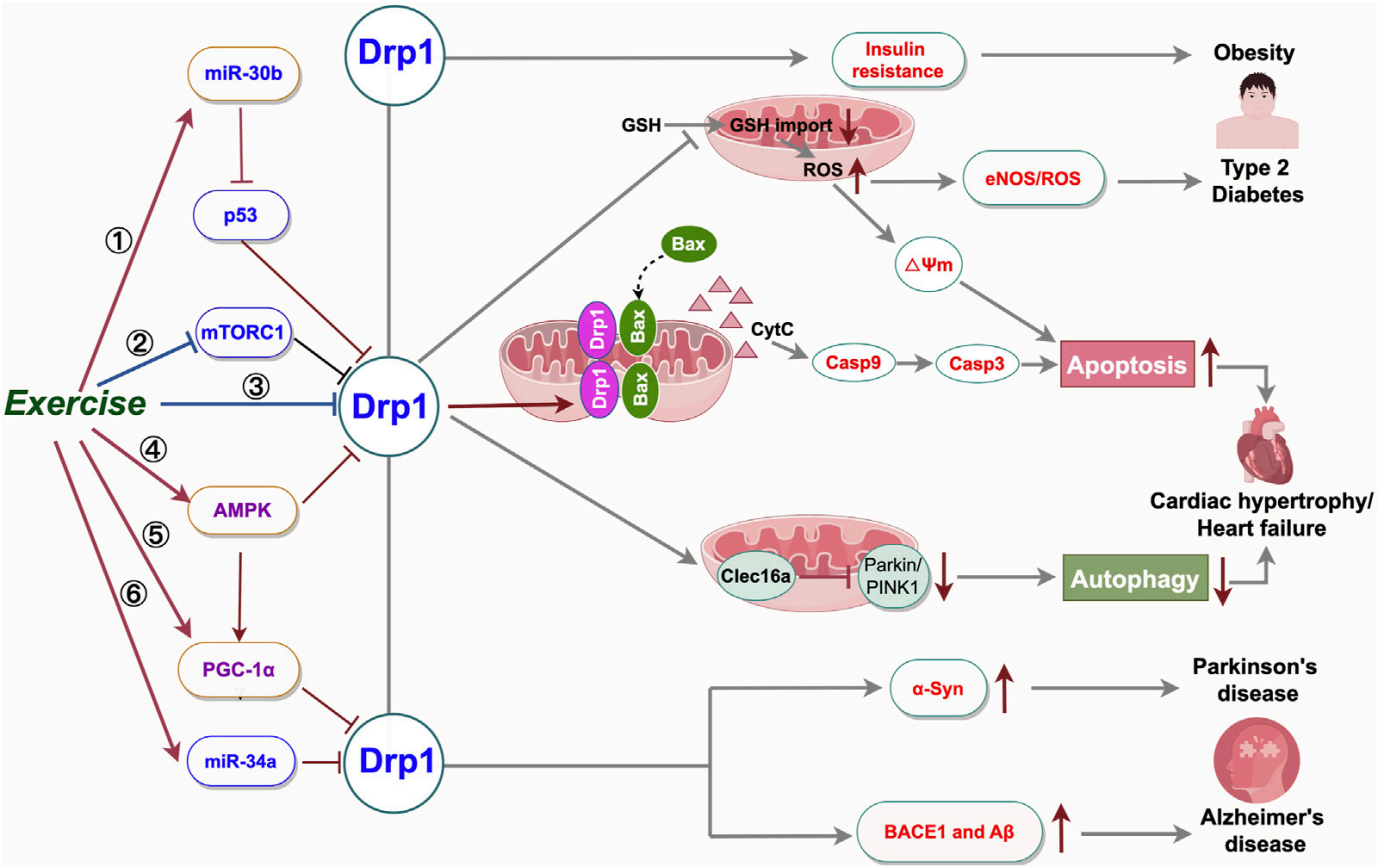
Summary of the mechanisms of physical exercise regulating Drp1-mediated mitochondrial fission to prevent and control mitochondrial dysfunction related diseases. High Drp1 levels alter cell signaling by increasing cell death, reducing mitochondrial autophagy, and disrupting metabolism. Exercise ameliorates common chronic diseases by modulating Drp1-related signaling targets and decreasing Drp1 activity. Targeted manipulation of the Drp1 pathway and its upstream controllers is poised to aid in treating disorders associated with abnormal mitochondrial fission. BACE1: β-amyloid precursor protein cleaving enzyme 1; Aβ: β-amyloid; α-Syn: α-synuclein; eNOS: endothelial nitric oxide synthase; ROS: Reactive Oxygen Species; miR-30b:microRNA 30b; miR-34a:microRNA 34a; GSH: glutathione. Export ID:YYTPUef00b

Recent research indicates that Drp1 activation prompts mitochondrial fission and elevates reactive oxygen species (ROS) levels, initiating apoptosis and inflammation. In pathologies such as atherosclerosis and heart failure, overactivation of Drp1 results in mitochondrial impairment and cellular damage. Phosphorylation, SUMOylation, and ubiquitination at Ser616 and Ser637 of Drp1 collectively modulate its activity and mitochondrial fission. Targeting Drp1 and its regulatory pathways offers novel strategies and therapeutic targets for treating diverse diseases. Aerobic exercise mitigates the onset and progression of prevalent chronic conditions, such as neurodegenerative diseases (e.g., Alzheimer’s, Parkinson’s, Huntington’s), cardiovascular diseases (e.g., coronary heart disease, heart failure), and metabolic disorders (e.g., type 2 diabetes, obesity), primarily by curbing Drp1-mediated excessive mitochondrial fission. Its benefits are mediated through modulation of key signaling pathway targets, as outlined above and depicted in Figure 3. In summary, aerobic exercise demonstrates significant therapeutic potential for common chronic diseases. Further research is required to clarify remaining issues.

Primarily, exercise interventions typically consist of moderate-intensity aerobic exercise, with the combination of aerobic and resistance training also demonstrating efficacy. However, the specific exercise modalities and intensities remain inconsistent. Hence, additional basic research is essential to compare and evaluate the impact of various exercise types and intensities on chronic disease improvement. Secondly, the mechanisms by which exercise influences target molecules in related signaling pathways, such as the effect of exercise on Drp1 activation states and its role in various disease models, require further investigation. Additionally, while most mechanistic studies are based on animal models, there is a paucity of clinical data. Consequently, more clinical trials are necessary to examine the therapeutic impacts of exercise and its mitochondrial-related mechanisms. Lastly, although exercise is a benign intervention, its effects can be limited in certain contexts. Future research should integrate exercise science, molecular biology, and clinical medicine in a multidisciplinary approach to comprehensively explore the benefits of exercise in chronic disease management.
